# Differences in factors influencing the use of eRehabilitation after stroke; a cross-sectional comparison between Brazilian and Dutch healthcare professionals

**DOI:** 10.1186/s12913-020-05339-7

**Published:** 2020-06-01

**Authors:** Berber Brouns, Leti van Bodegom-Vos, Arend J. de Kloet, Thea P. M. Vliet Vlieland, Ingrid L. C. Gil, Lígia M. N. Souza, Lucia W. Braga, Jorit J. L. Meesters

**Affiliations:** 1 Department of Orthopaedics, Rehabilitation and Physical Therapy, Leiden University Medical Center, Albinusdreef 2, 2333 ZA Leiden, The Netherlands; 2 Faculty of Health, Nutrition and Sports, The Hague University for Applied Sciences, The Hague, The Netherlands; 3 Basalt Rehabilitation, The Hague, /Leiden, The Netherlands; 4 Department of Biomedical Data Sciences, section Medical Decision Making, Leiden University Medical Center, Leiden, The Netherlands; 5 The SARAH Network of Rehabilitation Hospitals, Brasilia, Brazil

**Keywords:** Stroke, Barriers and facilitators, Implementation, Rehabilitation, eRehabilitation, Survey, Intercultural

## Abstract

**Background:**

To improve the use of eRehabilitation after stroke, the identification of barriers and facilitators influencing this use in different healthcare contexts around the world is needed. Therefore, this study aims to investigate differences and similarities in factors influencing the use of eRehabilitation after stroke among Brazilian Healthcare Professionals (BHP) and Dutch Healthcare Professionals (DHP).

**Method:**

A cross-sectional survey study including 88 statements about factors related to the use of eRehabilitation (4-point Likert scale; 1–4; unimportant-important/disagree-agree). The survey was conducted among BHP and DHP (physical therapists, rehabilitating physicians and psychologists). Descriptive statistics were used to analyse differences and similarities in factors influencing the use of eRehabilitation.

**Results:**

ninety-nine (response rate 30%) BHP and 105 (response rate 37%) DHP participated. Differences were found in the top-10 most influencing statements between BHP and DHP BHP rated the following factors as most important: sufficient support from the organisation (e.g. the rehabilitation centre) concerning resources and time, and potential benefits of the use of eRehabilitation for the patient. DHP rated the feasibility of the use of eRehabilitation for the patient (e.g. a helpdesk and good instructions) as most important for effective uptake. Top-10 least important statements were mostly similar; both BHP and DHP rated problems caused by stroke (e.g. aphasia or cognitive problems) or problems with resources (e.g. hardware and software) as least important for the uptake of eRehabilitation.

**Conclusion:**

The results indicate that the use of eRehabilitation after stroke by BHP and DHP is influenced by different factors. A tailored implementation strategy for both countries needs to be developed.

## Background

The rapid growth of digital health technology [1] provides efficient strategies for delivering rehabilitation while maintaining or improving effectiveness [2]. Therefore, it may offer a solution for the increasing need for care, especially in stroke rehabilitation, where incidence, survival rates and healthcare costs are growing [3]. Digital eRehabilitation programs offers an additional way of delivering conventional rehabilitation and can include physical and cognitive exercise programs, serious gaming, education [4–6] and e-consultations [7], delivered via a variety of information and communication technology (ICT) devices such as a computer, tablet and smartphone. eRehabilitation can be seen as an alternative way of providing all aspects of rehabilitation therapy, including intervention, maintenance activities, consultation, education, and training to clients at a remote location [4], and can included telerehabilitation (e.g. the provision of rehabilitation services to patients at a remote location using ICT), tablet-based therapy, and the use of commercially available devices like the Nintendo Wii [2, 4, 5, 8–11].

Randomized clinical trials and systemic reviews investigated the effects of eRehabilitation and showed multiple benefits of the use of eRehabilitation. eRehabilitation can decrease stroke-related impairments [5, 8, 9], relieve healthcare professionals from manual labour, make rehabilitation accessible to larger number of stroke patients [2], continue therapy-related cognitive and motor activities during and after discharge [4], decrease chronic disability during and after sub-acute rehabilitation, and facilitate home-therapy [10, 11]. Especially in regions with a paucity of socioeconomic resources and limited access to care, regions with the greatest burden of stroke worldwide [12], culturally-relevant eRehabilitation interventions are likely to be the most viable strategy to reduce burden [13].

However, the use of eRehabilitation in daily practice lacks worldwide [14] and the uptake of eRehabilitation is hamper by many factors. This included lack of confidence with hardware or software [15, 16], fear of losing social face-to-face contact [17, 18] and lack of meaningful reimbursement [7, 19]. In order to make eRehabilitation feasible, programs need to be tailored to the patients’ needs and sufficient support of a helpdesk for ICT is a prerequisite [20]. Studies performed in western countries concluded that eRehabilitation programs are generally considered feasible [5], however, in low- and middle income countries, future trails on the feasibility are needed [13]. Furthermore, it has been shown that eRehabilitation interventions need to address culture-specific issues in order to be effective [21], however, eRehabilitation interventions for patients are rarely culturally-adapted [22].

To improve the uptake of eRehabilitation after stroke, the identification of barriers and facilitators influencing this use is needed [22]. Most of the abovementioned research about barriers/facilitators in the use of eRehabilitation is performed in western countries (America, Canada, Australia, Europe), and as far as we know, no research is performed on the differences between western countries and other regions. Therefore, the aim of this paper is to describe the differences and similarities in factors influencing the use of eRehabilitation after stroke between Brazil and the Netherlands, countries with different cultures and healthcare systems.

## Methods

To identify differences and similarities in factors influencing the use of eRehabilitation after stroke between Brazilian and Dutch healthcare professionals, cross-sectional study conducted in a medical specialist rehabilitation setting involved a one-time cross-sectional online survey. This survey was developed based on the results of a preceding focus group study [23] and was conducted among Brazilian healthcare professionals (BHP) and Dutch healthcare professionals (DHP) working in stroke rehabilitation. The COREQ guidelines were used for adequate design of the focus groups [24] and STROBE statements were used for adequate sampling, analyses and reporting of the survey [25].

### Setting

#### Brazil

Brazil has 209 million inhabitants, of which 70% has internet access. Brazil has a population density of 25 inhabitants/km^2^ and gross domestic product of 8.2 US dollar/inhabitant. Data from a national prospective study indicate an annual incidence of 108 cases per 100,000 inhabitants. Stroke Care Guidelines are established involving pre-hospital treatment, intervention in acute stroke, and follow-up at rehabilitation centres [26, 27]. Rehabilitation can take place on an outpatient basis, an inpatient basis, or during hospitalization. In all settings, interventions are delivered by multidisciplinary teams working in an interdisciplinary manner with active patient participation and family inclusion. Specialized professionals include physicians, nurses, social workers, physical therapists, occupational therapists, speech therapists, psychologists, hospital educators, physical education instructors, and nutritionists. The treatment and rehabilitation process are free of charge; the national health budget covers all costs.

#### Netherlands

The Netherlands have 17 million inhabitants, of which 95% has internet access. The Netherlands has a population density of 507 inhabitants/km^2^ and a gross domestic product of 56.4 US dollar/inhabitant. The annual incidence of stroke in the Netherlands was estimated 107 cases per 100,000 inhabitants [28]. Incidence and mortality rates decline as a result of better and faster treatment [29] and stroke burden in terms of the absolute number of people affected by stroke increase [30]. About 10% of the stroke survivors follow multidisciplinary in or out-patient rehabilitation in a medical specialist rehabilitation setting [31], including physiotherapy, speech therapy, occupational therapy, psychology and a social worker, coordinated by a rehabilitation physician [32]. A rehabilitation plan is made and evaluated during weekly team meetings, and patients and family are involved if needed. Rehabilitation consisted of individual and group exercise [32]. Six months after stroke, on average 60% of the patients are community living again [33]. Most costs are reimbursed by the healthcare insurance provider, with out of pocket costs for the patients of maximum €885,-.

### Study population

Inclusion criteria for both BHP and DHP were 1) at least 2 years of working experience in a multidisciplinary stroke team and 2) still actively treating stroke patients. Invited BHP included neurologists, physical therapists, occupational therapists, psychologists, nurses, social workers, speech therapists, hospital educators, and physical educators from the SARAH Network of Rehabilitation Hospitals. BHP working with stroke patients were invited via internal communication within SARAH, a network that has nine rehabilitation centres throughout Brazil. Invited DHP included rehabilitation physicians, psychologists and physical therapists. DHP were identified using a Dutch medical address book including contact information of most healthcare professionals in the Netherlands, across the country. Since the participating countries are geographically far apart from each other, it was esteemed unlikely that one person could receive both the Brazilian and Dutch invitation, but this is not impossible. All eligible healthcare professionals (both Brazilian and Dutch) received an invitation email including a link to the online survey, in Dutch to the DHP (June 2017) and in Portuguese to the DHP (October 2017). Non-responders received two reminders, first after 2 weeks and second after 4 weeks that and the survey was available for 5 months.

### Survey development and content

To develop the survey, eight focus groups were organized with both patients/informal caregivers and healthcare professionals (details about the analysis and results are published elsewhere [23]). Focus groups were used to collect a broad spectrum of possible factors influencing the use of eRehabilitation, including attitudes, experiences and expectations of the healthcare professionals [34]. In this, eRehabilitation is the use of ICT to deliver conventional rehabilitation care and can be used to support therapy related activities, like physical and cognitive exercises, education and communication. Thirteen DHP working in stroke rehabilitation participated, including rehabilitation physicians (*n* = 4, 31%), physical therapists (*n* = 3, 23%), occupational therapists (*n* = 3, 23%), psychologists (*n* = 1, 8%), speech therapists (*n* = 1, 8%), and managers (*n* = 1, 8%).

All focus groups were audiotaped and transcribed in full in Dutch. The transcripts were qualitatively analysed using directed content analysis, in which the researchers used a theory or relevant research findings as guidance for initial code [35], in this case the implementation model of Grol and Wensing [36]. This model was chosen because it provides a framework for identifying and categorizing factors that influence the use of innovations in healthcare [36]. A total of 88 barriers/facilitators that impact the use of eRehabilitation were identified. Those were grouped into fourteen factors, divided at five levels of Grol (see Table 1); the innovation (e.g. content of eRehabilitation, feasibility, accessibility), the organisational context (e.g. tasks and responsibilities of involved end-users, time and resources), the individual patient (e.g. skills, knowledge, motivation the change and patient characteristics), the individual professional (e.g. skill, knowledge, motivation to change) and the financial arrangements (e.g. insurance).

**Table 1:** Results of focus groups; factors influencing the use of eRehabilitation (2 focus groups)

Level	Factor	Sub-factor
Innovation	Accessibility	Time frame in which eRehabilitation is accessible
		Devices on which eRehabilitation is accessible
	Feasibility	Helpdesk function
		Tailored to patients’ situation
	Attractiveness	Ease of use of eRehabilitation
		Content of eRehabilitation program
	Privacy	Privacy and safety of patient data
	Advantages of use	Added value of innovation offered
Organizational context	Organization of care	Tasks and responsibilities healthcare professional
		Tasks and responsibilities informal caregiver
		Tasks and responsibilities organization
	Resources	Software
		Hardware
	Time	Time
Individual patients	Motivation to change	Reasons to use eRehabilitation for patients
	Motivation not to change	Reasons not to use eRehabilitation for patients
	Patient characteristics	Impairments after stroke
Individual professional	Motivation to change	Reasons to use eRehabilitation
	Motivation not to change	Reasons not to use eRehabilitation
Economic & political context	Financial arrangements	Insurance

To prioritize all barriers/facilitators identified in the focus groups, a survey was conducted in the Netherlands (June 2016) and Brazil (December 2017). The survey included questions about personal characteristics and statements about barriers/facilitators influencing the use of eRehabilitation. The questionnaire (Additional file 1) was specifically developed for an overarching research project to identify factors influencing the use of eRehabilitation. Results of a study concerning only Dutch responses were published elsewhere [20].

#### Socio-demographic-, disease- and work-related characteristics

The survey started with the question ‘Are you working with stroke patients?’ If not, the survey was ended. If ‘yes’, 12 questions followed regarding age, gender, work setting (primary care/rehabilitation centre/general hospital), years of work experience, number of new stroke patients per month and their current use of eRehabilitation (no, yes; if yes: exercises/games/information).

#### Influencing barriers/facilitators

Each potential barrier/facilitator identified in the focus group study was translated into a neutral statement. A total of 88 statements were formulated based on the transcripts of the focus groups. The influence for the use eRehabilitation of each statement was rated on a 4-point Likert scale (1 = unimportant, 2 = somewhat unimportant, 3 = somewhat important, 4 = important or 1 = disagree, 2 = partly disagree, 3 = partly agree, 4 = agree).

The survey was tested in a pilot among three DHP (2 males, 2 physical therapists, 1 occupational therapist, mean age 38 years old, mean working experience 13.3 years). The survey was tested for feasibility, legibility, readability and presentation (e.g., perceived statement difficulty, response errors, etc.). Testing led to small changes in the phrasing and layout.

The survey was based on the results focus groups in the Netherlands and developed in Dutch. For the BHP, the survey was translated by a qualified Portuguese-language translator. First, the Dutch version was translated into English by the translation agency *Attached Language* and the translation was discussed in the project team leading to minor changes. Subsequently, the English version was translated into Portuguese and was tested by two Portuguese project members. Differences were discussed and adaptations were made in three rounds until the Portuguese questionnaire was similar to the original Dutch version.

### Data analysis

Participants who completed > 90% of the survey were included in the analysis, which was executed using Statistical Packages for the Social Sciences (IBM SPSS 22.0), and no imputations were done for missing data. Personal characteristics were analysed using descriptive statistics. T-test or Pearson Chi-square test was used to compare age, gender, number of new patients, work experience and the use of eRehabilitation between BHP with DHP.

Based on the median score, all statements influencing the use of eRehabilitation were given a ranking (lowest number equals large influence), separately for the BHP and DHP. For the statements with a similar median, definite ranking was based on the mean. The top-ten most and least influencing statements were noted and differences in ranking were calculated to describe the level of agreement among DHP and BHP. The ranking of all statements for both the DHP and BHP were plotted on a scatterplot, including a 95% confidence interval (CI). Additionally, these analyses were performed with only the disciplines included both in the Netherlands and Brazil (i.e. physical therapists, psychologists and physicians).

### Ethical issues and approval

All participants gave written informed consent prior to participation. Data were collected and analysed anonymously. This study was approved by the Medical Ethical Review Board of the Leiden University Medical Centre [P15.281] and the Medical Ethics Board of SARAH Network of Rehabilitation Hospitals.

## Results

### Study population

**Fig. 1:**
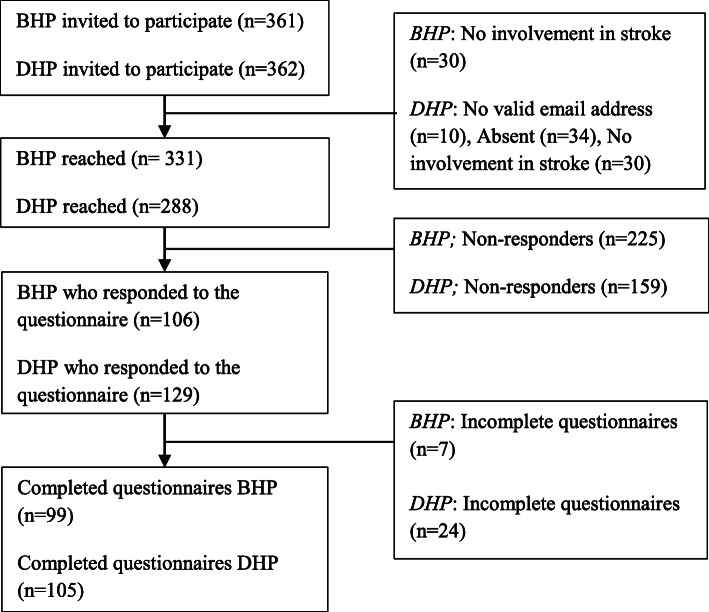
Flowchart

Of the 361 invited BHP, 331 were reached and 99 responded (response rate 30%); of the 362 invited DHP, 288 were reached and 105 responded (response rate 37%). Thirty (8.3%) of the BHP and 30 (10%) DHP did not work with stroke patients and were therefore excluded from the analyses (see Fig. 1). Table 2 shows that BHP and DHP did not differ significantly in age (40.0 (SD 6.4) and 42.0 (SD 10.5) years old, respectively), gender (*n* = 21 (21%) and *n* = 25 (24%) male, respectively), work experience (15.6 (SD6.2) and 14 (SD10) years, respectively) and previous use of eRehabilitation (*n* = 50 (50%) and *n* = 40 (38%) respectively). BHP had significantly more new patients each month compared to the DHP (*p* = 0.00). DHP included physical therapists (*n* = 41, 39%), psychologists (*n* = 14, 13%) and physicians (*n* = 47, 45%), BHP included physical therapists (*n* = 14, 14%), psychologists (*n* = 12, 12%), physicians (*n* = 10, 10%); additionally, nurses (*n* = 28, 26%), hospital educators (*n* = 3, 3%), physical education teachers (*n* = 10, 10%) and neurologists (*n* = 5, 5%) were included in the BHPs.

**Table 2:** Characteristics of Brazilian and Dutch healthcare professionals participating in the survey study

Characteristics	BHP (*n* = 99)	DHP (*n* = 105)
Age, years (mean, SD)	40.0 (6.4)	42.0 (10.5)
Sex, (n male, %)	21 (21)	25 (24)
Work experience, years (mean, SD)	15.6 (6.2)	14.0 (10.0)
Number of new patients per month (mean, SD)	**13.5 (9.5)**	**8.0 (8.9)**
Discipline, (n, %)		
Physical therapist	14 (14)	41 (39)
Psychologist	12 (12)	14 (13)
Physician	10 (10)	47 (45)
Nurse	28 (26)	.
Occupational therapist	3 (3)	.
Hospital-based educator	3 (3)	.
Physical education instructor	10 (10)	.
Neurologist	5 (5)	.
Other*	14 (14)	3 (3)
Work setting** (n, %)		
Health centre in primary care	.	10 (10)
Rehabilitation centre	97 (97)	75 (71)
Hospital	4 (4)	34 (32)
Use of digital rehabilitation tools (n yes, %)	50 (50)	40 (38)

*BHP*; Brazilian healthcare professional, *DHP*; Dutch healthcare professional

In bold significant differences between BHP and DHP (*p* = 0.00)

* Occupational therapist, Speech therapist, Nutritionist, Social worker, **Multiple answers possible

### Most and least influencing statements

Tables 3 and 4 show the ten most and ten least influencing statements for DHP and BHP to use eRehabilitation after stroke. In the top-10 most influencing factors, four statements were found for both BHP and of DHP, and twelve statements were found in the top-10 of only one group (see Table 3). The six statements found for only BHP were related to the factor Patient Motivation to Change (i.e., improved therapy adherence and health outcomes) and the Organization of Care (i.e., sufficient time and support from the organization); the six statements found for only DHP were mostly related to the factor Feasibility of eRehabilitation (like a helpdesk and support).

**Table 3:** Statements with the most influence on the use of eRehabilitation

Statement I would use e-rehabilitation, if…	Factor	Barrier/ facilitator	Brazil (***n*** = 99)		Netherlands (***n*** = 105)	
			Median (IQR)	Ranking	Median (IQR)	Ranking
It contributes to the patient’s therapy compliance	Patient motivation to change	F	4 (4–4)^a^	1	4 (4–4)	8
eRehabilitation has a positive influence on recovery	Patient motivation to change	F	4 (4–4)^a^	2	4 (4–4)	2
I can tailor the content of eRehabilitation to the patient’s personal situation	Feasibility	F	4 (4–4)^a^	3	.	12
I have time to (learn to) use eRehabilitation	Organization of care	F	4 (4–4)^a^	4	.	21
I feel supported from within the organization to use eRehabilitation	Organization of care	F	4 (4–4)^a^	5	.	32
eRehabilitation offers a way to independently continue therapy after discharge	Patient motivation to change	F	4 (4–4)^a^	6	.	15
ICT-problems are solved directly	Organization of care	F	4 (4–4)^a^	7	4 (4–4)	7
Logging on is easy	Accessibility	F	4 (4–4)^a^	8	4 (4–4)	3
My patient wants to use eRehabilitation	Patient motivation to change	F	4 (4–4)^a^	9	.	11
Exercises to train cognitive functioning^b^	Attractiveness	F	4 (4–4)^a^	10	.	55
A helpdesk is available for patients	Feasibility	F	.	13	4 (4–4)	1
Video instructions on how to use e-rehabilitation are available for patients	Feasibility	F	.	17	4 (4–4)	4
A menu with frequently asked questions (FAQ) for patients	Feasibility	F	.	21	4 (4–4)	5
The patient can read information about stroke	Feasibility	F	.	19	4 (4–4)	6
Decisions made during a consult are documented and visible for patients^b^	Advantage of Use	F	.	67	4 (4–4)	9
Insights in goals that are achieve	Attractiveness	F	.	24	4 (3–4)	10

· = no part of most influencing statements, *B* Barrier, *F* Facilitator, *IQR* Interquartile range

**Fig. 2:**
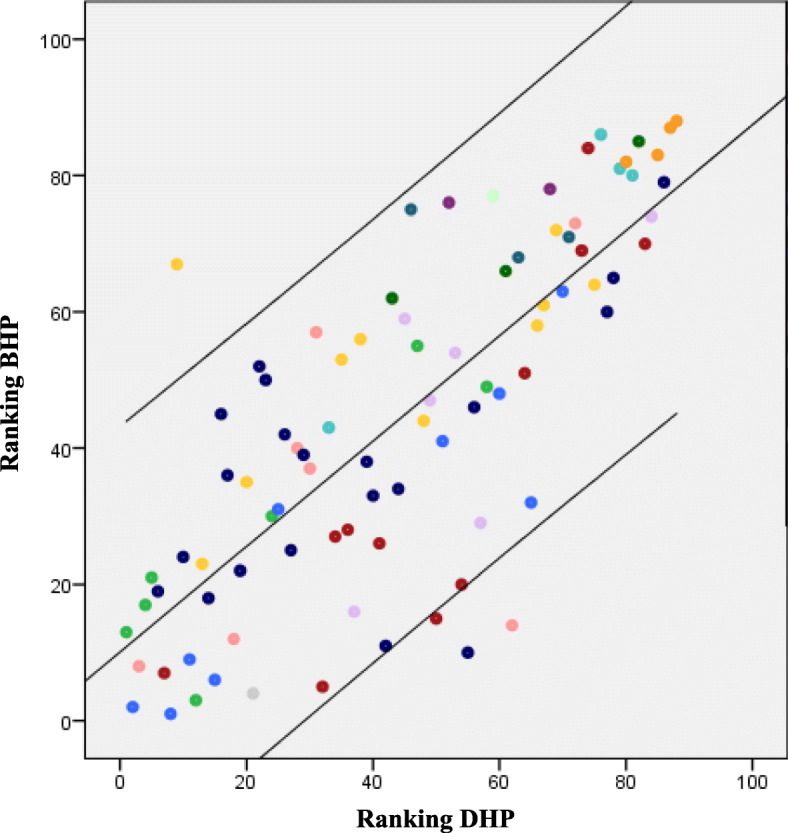
Scatterplot of the ranking of all statements for the Brazilian healthcare professionals (BHP) and Dutch healthcare professionals (BHP). Lower values are statements with more influence.

^a^In the top-ten when only physical therapists, rehabilitation physicians and psychologist are included^b^ Outside 95%Confidence interval in scatterplot, see Fig. 2

**Table 4:** Statements with the least influence on the use of eRehabilitation

Statement I would not use e-rehabilitation, if…	Factor of Grol	Barrier/ facilitator	Brazil (***n*** = 99)		Netherlands (***n*** = 105)	
			Median (IQR)	Ranking	Median (IQR)	Ranking
The patient has too many physical disabilities after stroke	Patient characteristic	B	2 (1–2)^a^	88	1 (1–2)	88
The patient has too much aphasia after stroke	Patient characteristic	B	2 (1–2)^a^	87	2 (1–2)	87
I believe that there will be problems with software	Resources	B	2 (1–3)^a^	86	.	76
There is too little scientific evidence for the effectiveness of eRehabilitation	Professional motivation not to change	B	2 (1–3)^a^	85	2 (2–3)	82
Implementation of eRehabilitation happened simultaneously with other ICT projects	Organization of care	B	2 (2–3)^a^	84	.	74
The patient has too many cognitive disabilities after stroke	Patient characteristic	B	2 (2–3)^a^	83	2 (2–3)	85
The patient has visual problems	Patient characteristic	B	2 (2–3)^a^	82	2 (2–3)	80
Problems with the devices on which eRehabilitation is used	Resources	B	3 (1–4)	81	3 (1–3)	79
Problems with the internet connection	Resources	B	3 (1–4)	80	3 (1–3)	81
The patient cannot compare his/her results with the scores of other stroke patients	Attractiveness	F	3 (2–3)^a^	79	2 (1–3)	86
I cannot compare patients results with the scores of other stroke patients	Attractiveness	F	.	74	2 (2–3)	84
The healthcare professional contacts the patients if he/she exercises too little	Organization of care	F	.	70	2 (2–3)	83

· = no part of least influencing statements, B; barrier, F; facilitator, IQR; Interquartile range

^a^In the top-ten when only physical therapists, rehabilitation physicians and psychologist are included

On the other hand, the statements that BHP and DHP considered not influencing the use of eRehabilitation were comparable, with eight statements found in the top-10 of BHP and DHP. Factors that did not influence eRehabilitation use were related to the factor Patient characteristics (i.e., cognitive and physical disability or aphasia) and the factor Resources (i.e., problems with the internet connection or hard- and software).

The abovementioned analyses were also performed including only the disciplines that were represented in both countries (i.e. physical therapists, rehabilitation physicians and psychologists), resulting in comparable findings. Only the two statements ‘*Problems with the devices on which eRehabilitation is used*’ and ‘*Problems with the internet connection’* were not found in the top-ten least influencing statements of this sub-analysis; the top-ten most influencing statements was fully comparable with the results of the all respondents (see Tables 3 and 4).

### Difference and similarities in ranking

The difference in ranking for the BHP and DHP was calculated for each statement (Additional file 1). The mean absolute difference in ranking between BHP and DHP was 11.2 (SD 15.9, range 0–58). In Fig. 2, the ranking of the Brazilian responses is plotted against the Dutch responses. Four statements were found outside the 95% CI. BHP reported the following statements more frequently as important than DHP: 1) *‘The eRehabilitation program is accessible offline’,* 2) *‘Exercises to train cognitive functioning’* and 3)*. ‘eRehabilitation is used by the entire multidisciplinary team’*. DHP reported the following statement more frequently as important than BHP: ‘*Decisions made during a consult are documented and visible for patients*.’ Two of those statements (the second and fourth) were found in the top-10 most influencing statements of respectively BHP and DHP (see Table 3).

For the majority of the factors, the statements constituting that factor were spread out on a broad range of the scatterplot, with at least one statement within the 20 most and one statement in the 20 least influencing statements (Additional file 1 and Fig. 2). Only the statements constituting the factors Resources, Patient Motivation not to change and Patient characteristics were found only with a low influence.

## Discussion

In this study, we investigated differences and similarities in factors influencing the use of eRehabilitation after stroke among healthcare professionals from Brazil and the Netherlands. The statements with the highest influence on the use of eRehabilitation differed between BHP and DHP; BHP agreed more with factors related to the benefits for the patients and organizational constrains, DHP agreed more with factors related to the feasibility of the use of eRehabilitation. The statements with the least influence on the use of eRehabilitation were comparable for BHP and DHP, and were related to patient characteristics and resources. This means that BHP and DHP indicate that the use of eRehabilitation is influenced by different factors and tailored implementation strategies for both countries need to be developed separately [22].

For BHP, and with a lesser frequency for DHP, the factor Motivation to change was important. Benefits of the use of eRehabilitation were found important before, including the possibility to train at home [37], independently continue therapy activities [4] and easily accessible contact with a healthcare professionals after discharge or during outpatient therapy [17, 38]. For BHP, time and support for the healthcare professional from the organization is also important. Facilitating conditions, including time, communication and education, was found to be an important facilitating factor in the use of eRehabilitation after stroke before [38, 39]. For DHP, a thorough helpdesk delivering support for patients and healthcare professional is crucial. This is in line with a review of Pugliese (2018) concluding that the most reported patient barrier was following instructions about how to use the device [40].

Concerning the content of the eRehabilitation intervention, for the BHP speech and cognitive exercisers are important, were the DHP focus on physical exercises, and offline accessibility seems important in Brazil but not in the Netherlands. For the DHP it is important that decisions that were made during a consult are incorporated in the eRehabilitation intervention. Therefore it can be concluded that not only the implementation strategy should be adapted to the wishes of the end-users [17], but also the eRehabilitation intervention.

Most factors were constructed of statements that were spread over a broad ranking and included both statements influencing and non-influencing the use of eRehabilitation. So some differences might remain hidden at factor level, since statements within a factor compensate for each other, differences can be found at statement levels. Therefore, it is important to investigate barriers/facilitators for the implementation of eRehabilitation in detail rather than on the level over overarching factors.

Although our study revealed some important differences and similarities among Brazilian and Dutch healthcare professionals, the results have to be interpreted with care due to some limitations. First, only 36% of the BHC were physical therapists, psychologists and rehabilitation physicians; i.e. the disciplines invited in the Netherlands. However, when only the responses of the Brazilian physical therapists, psychologists and rehabilitation physicians were taken into account, the results of the analyses were comparable with the results of all BHPs. Therefore, it seems plausible that differences are caused by the various contexts and not by the specific professional backgrounds of the respondents. Second, the response rate of 30–37% in our study may have led to response bias because those who responded to the invitation to participate in the survey were probably more interested in eRehabilitation. As a consequence, the perspective of end-users with less interest in and experience with eRehabilitation might be missing. A third limitation is that the survey statements were based on the results of focus groups performed in the Netherlands. Consequently, we might have missed factors influencing the use of rehabilitation in Brazil that are not present in the Netherlands. However, the developed survey covered all levels of the framework of Grol and showed high amount of saturation (e.g. for two consecutive focus groups, no new factors were found), which reduces the chance of missing potentially important factors. At last, the generalizability of our results beyond the Netherlands and Brazil may be limited. The countries involved differed a lot on important factors (e.g. income and demographics), which is crucial for the development of a successful implementation strategy. It may be assumed that other counties will differ as well, which should be further investigated.

## Conclusion

Important differences were found in factors influencing the use of eRehabilitation after stroke between BHP and DHP. For BHP, the use of eRehabilitation after stroke was most influenced by support from the rehabilitation organization and the potential benefits of the use of eRehabilitation. For DHP, the feasibility of the use of eRehabilitation for the patient was most influential. Implementation strategies should incorporate those differences, including an eRehabilitation intervention adapted to the wishes of the end-users. Statements with low influence, such as problems caused by patient characteristics after stroke or problems with resources, were comparable for both groups and should have less priority in the implementation strategies. More research about differences between disciplines in Brazil and the generalizability of those results for other countries is needed.

## Supplementary information

**Additional file 1.** Ranking of the importance of the statements based on the median and mean, for Brazilian and Dutch healthcare professionals

## Abbreviations

ICT: Information and communication technology
BHP: Brazilian healthcare professionals
DHP: Dutch healthcare professionals
