# Correction to: Differences in factors influencing the use of eRehabilitation after stroke; a cross-sectional comparison between Brazilian and Dutch healthcare professionals

**DOI:** 10.1186/s12913-020-05457-2

**Published:** 2020-07-01

**Authors:** Berber Brouns, Leti van Bodegom-Vos, Arend J. de Kloet, Thea P. M. Vliet Vlieland, Ingrid L. C. Gil, Lígia M. N. Souza, Lucia W. Braga, Jorit J. L. Meesters

**Affiliations:** 1 Department of Orthopaedics, Rehabilitation and Physical Therapy, Leiden University Medical Center, Albinusdreef 2, 2333 ZA Leiden, The Netherlands; 2 Faculty of Health, Nutrition and Sports, The Hague University for Applied Sciences, The Hague, The Netherlands; 3 Basalt Rehabilitation, The Hague/ Leiden, The Netherlands; 4 Department of Biomedical Data Sciences, section Medical Decision Making, Leiden University Medical Center, Leiden, The Netherlands; 5 The SARAH Network of Rehabilitation Hospitals, Brasilia, Brazil

**Correction to: BMC Health Serv Res 20, 488 (2020)**

**https://doi.org/10.1186/s12913-020-05339-7**

Following the publication of the original article [1], it was noted that due to a typesetting error the legend in the Fig. 2 is missing.

The correct figure has been included in this correction, and the original article has been corrected.

**Fig. 2:**
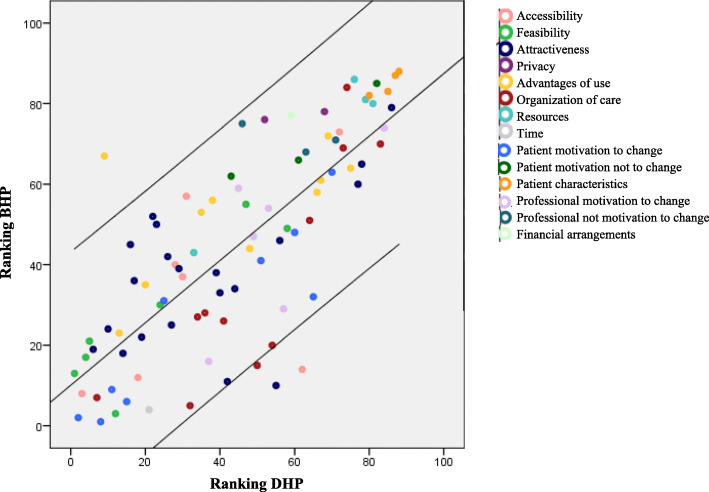
Scatterplot of the ranking of all statements for the Brazilian healthcare professionals (BHP) and Dutch healthcare professionals (BHP). Lower values are statements with more influence

## References

[1] Brouns (2020). Differences in factors influencing the use of eRehabilitation after stroke; a cross-sectional comparison between Brazilian and Dutch healthcare professionals. BMC Health Serv Res.

