# Supplementary material for: Differences in factors influencing the use of eRehabilitation after stroke; a cross-sectional comparison between Brazilian and Dutch healthcare professionals
Source: BMC Health Serv Res. 2020 Jun 1;20:488. doi: 10.1186/s12913-020-05339-7 (PMC7268386; doi:10.1186/s12913-020-05339-7)
Supplement: Supplementary file 1 — Additional file 1. Ranking of the importance of the statements based on the median and mean, for Brazilian and Dutch healthcare professionals [file 12913_2020_5339_MOESM1_ESM.pdf]

Additional file 1

Ranking of the importance of the statements based on the median and mean, for Brazilian and Dutch healthcare professionals

**Level**	Factor	Statement	**Rank-ing BHP**	**Rank-ing DHP**	**Differ-ence**
**Innovation**	Accessibility (n=7)	The eRehabilitation program is accessible for a certain period	73	72	1
		Logging in is easy	8	3	5
		The possibility to use eRehabilitation on all devices (i.e.tablet, smartphone)	12	18	6
		Patients' training results are accessible for a healthcare professional	37	30	7
		The use of eRehabilitation does not result in many screens	40	28	12
		The eRehabilitation program is accessible without login in every time	57	31	26
		The eRehabilitation program is accessible offline	14	62	48
	Feasibility (n=7)	A helpdesk via telephone or mail is available for healthcare professionals	30	24	6
		A menu with frequently asked questions (FAQ)for healthcare professionals	55	47	8
		Instruction videos explaining healthcare professionals how to use eRehabilitation	49	58	9
		The content of eRehabilitation can be tailored to the patients' situation	3	12	9
		Helpdesk via telephone or email is available for patients	13	1	12
		Instructions videos explaining how to use eRehabilitation for patients	17	4	13
		A menu with frequently asked questions (FAQ) for patients	21	5	16
	Attractiveness (n=20)	Insight in what is trained online	38	39	1
		Insight in training results online	25	27	2
		A module about how to deal with stroke (psycho-education)	22	19	3
		The possibility for patients to read information about patient association	18	14	4
		Comparing the training results with other stroke patients	79	86	7
		Tests giving insight in the recovery after stroke	33	40	7
		Track physical activities (like walking and sitting) with a device	46	56	10
		Insight in how many is trained online	39	29	10
		Step-by-step explanation of daily activities (e.g. laying the table)	34	44	10
		Keep track of the body weight	65	78	13
		The possibility for patients to read information about stroke	19	6	13
		Insights in goals that are achieve	24	10	14
		Insight in the amount of physical activity (including duration) online	42	26	16
		Keep track of heart rate	60	77	17
		Links to website with relevant information about stroke for patients	36	17	19
		The possibility to contact other stroke patients	50	23	27
		Exercises to train physical functioning	45	16	29
		The possibility for informal caregiver to contact other informal caregivers	52	22	30
		Speech exercises for patients with aphasia	11	42	31
		Exercises to train cognitive functioning	10	55	45
	Privacy (n=2)	A safety label for digital rehabilitation programs like eRehabilitation	78	68	10
		Data safety when sending information and training results from the home address to the rehabilitation center	76	52	24
	Advantage of use (n=10)	Video calling for contact between patient and healthcare professionals (e-consult)	72	69	3
		An agenda including time for planned exercises	44	48	4
		An agenda including the possibility to ask for an appointment with a healthcare professional	61	67	6

**Innovation** (*continuation*)	Advantage of use (*continuation*)	The possibility to make videos of performing exercises, so the execution can be assessed by the healthcare professional	58	66	8
		An agenda including reminders for planned appointments and tasks	23	13	10
		An agenda including the possibility to make and administer an appointment with a healthcare professional	64	75	11
		An agenda including appointments with the healthcare professionals	35	20	15
		Insight in the final reports about the rehabilitation results	56	38	18
		Completing questionnaires that give insight in the recovery after stroke	53	35	18
		Decisions made during a consult are documented and visible for patients	67	9	58
**Organizational context**	Organization of care (n=11)	ICT-problems are solved directly	7	7	0
		Possibility for the healthcare professionals to check if exercises are performed	69	73	4
		Setting up goals of the rehabilitation therapy with the healthcare professionals	27	34	7
		Evaluating goals of the rehabilitation therapy with the healthcare professionals	28	36	8
		The implementation of eRehabilitation coincides with implementation of other ICT-projects	84	74	10
		The healthcare professional contacts the patients if he/she exercises too little	70	83	13
		The healthcare professional watches video to assess if exercises are perform correctly at home	51	64	13
		Discussing training results with the healthcare professional during a consult	26	41	15
		I feel supported from within the organization to use eRehabilitation	5	32	27
		Ambassadors (forerunners) in the form of direct colleagues who can answer questions about eRehabilitation	20	54	34
		eRehabilitation is used by the entire multidisciplinary team	15	50	35
	Resources (n=4)	Problems with the internet connection at home	80	81	1
		Problems with the devices on which eRehabilitation is used	81	79	2
		There is no need to download special programs to use eRehabilitation	43	33	10
		Problems with the software of eRehabilitation	86	76	10
	Time (n=1)	I have sufficient time to (learn to how to) use eRehabilitation	4	21	17
**Individual patient**	Motivation to change (n=9)	Training with eRehabilitation has a positive influence on recovery	2	2	0
		My patient wants to use eRehabilitation	9	11	2
		eRehabilitation offers variation in exercises	31	25	6
		eRehabilitation contributes to the therapy adherence	1	8	7
		I can ask my healthcare professionals questions about my training results online	63	70	7
		eRehabilitation offers a way to independently continue therapy after discharge	6	15	9
		Reduced travel time since eRehabilitation offers the possible to exercise at home	41	51	10
		Exercises in which it is possible to win or get points (serious games)	48	60	12
		eRehabilitation offers an easy way to contact a professional again after discharge	32	65	33
	Motivation not to change (n=3)	There is little scientific evidence for the effectiveness of eRehabilitation	85	82	3
		I give patients false hope that the continuation of exercising is useful while it is not	66	61	5
		Less contact between patients because they practice at home more often	62	43	19

	Patient characteristics(n=4)	The patient has physical problem	88	88	0
		The patient has aphasia	87	87	0
		The patient has cognitive problems	83	85	2
		The patient has visual problems	82	80	2
		Insight in how much a patient has trained	54	53	1
		Insight in the training results of a patient	47	49	2
		The results of the patient can be compared with the results of other stroke patients	74	84	10
**Individual Professional**	Motivation to change (n=6)	Possibility to see what activities a patient has done during a day (including time)	59	45	14
		Insight in the patient achieving set goals	16	37	21
		Insight in what a patient has trained	29	57	28
	Motivation not to chan-ge(n= 3)	I have less direct contact (face-to-face) with my patient	71	71	0
		My therapy is replaced by eRehabilitation	68	63	5
		Time for using eRehabilitation is at the expense of therapy time with the patient	75	46	29
**Eco & pol**	Fin. Ar. (n=1)	The use of eRehabilitation is not reimbursed by the health insurance	77	59	18

BHP; Brazilian Healthcare Professionals; DHP; Dutch Healthcare professionals; Eco & pol; Economical and political context. Fin. Ar.; Financial arrangements
